# The Hospitals of the Women’s Homœopathic Association

**Published:** 1887-05

**Authors:** Walter M. James


### THE HOSPITALS OF THE WOMEN'S HOMŒOPATHIC ASSOCIATION OF PENNSYLVANIA.

The above-named Institution, located at Twentieth Street and Susquehanna Avenue, Philadelphia, has had public attention turned upon it very particularly during the past month by reason of the bold action taken by eight of the lady physicians upon the visiting staff.

They have held a caucus, in which they have drawn up a written protest and appended to it their resignations from the staff.

The allegation is that the Board of Managers, not one of whom is a physician, has audaciously interfered with the plan of treatment of these doctors, interfered with their "potencies and remedies," and refused to permit the use of alcoholic liquors, and also of Carbolic Acid as a disinfectant, thus hampering their action in relieving patients and endangering their lives, whilst the responsibility is not removed from the physicians.

The discontent of these women doctors has been freely ventilated in the daily newspapers of this city. They have laid their complaints before the County Society, which organization has indorsed their action in resigning, and the indorsement is published in the daily papers. In his valedictory oration before the graduating class of Hahnemann Medical College, Dr. Betts has alluded to the affair thus: "In our hospitals, where the necessity of disinfection is recognized, you find that all the best means known to science are employed to prevent the spread and propagation of diseases, and where a misguided sentiment would restrict our physicians from using these agents, they refuse to be held responsible for the results and withdraw from the institution."

In justice to the Managers, and by reason of the wide publicity given to the affair by these doctors, we are compelled to state their side of the case.

The Woman's Hospital is governed by a set of rules. These rules are published, and are therefore well known. Physicians who take service in this Hospital are understood to subscribe to these rules. When they can no longer feel themselves bound by them they are expected to resign.

The lady physicians in this case intended to depart from the letter as well as the spirit of these rules, and when reminded of their delinquency practically and sensibly acknowledged their error by resigning. This is the whole case in a nutshell.

If it be asked in what way they ignored these rules, we may state that crude drugs were brought into the Hospital for common use; it was desired to administer cathartics and Persulphate of Iron, when rule first distinctly says: "No medicines, except strictly homœopathic potentized remedies, shall be allowed for use in the dispensaries or in any department of the Hospital." In their protest these ladies justify this practice by quoting from a recent lecture delivered by the venerable Dr. Lippe within the walls of the Hospital, in which he says: "The whole scale, from the crude natural substances up to the higher and highest infinitesimals, should be open to the choice and the practice of every sensible and candid person."

This statement, sensible and innocent enough in itself, is deliberately perverted to justify the use of *massive* doses, which are contrary to the spirit and letter of the homœopathic teaching, and utterly opposed to its plain philosophy. Thus perverted, it constitutes the argument, old and threadbare, used in the past by men who were avowed mongrels, to justify all sorts of allopathic procedures, to the scandal of the true followers of Hahnemann, and the delight of the cynical regulars, who extract from it all the arguments they need against the new school.

It is the argument used a few years ago by a well-known physician and professor in New York, when he arrogantly declared, in the meeting of the County Society, that he gave purgatives, diuretics, etc., and "considered himself a very good homœopath," thereby provoking the New York *Medical Record* to the brilliant and pungent remark that "Homœopathy had committed suicide in the open streets."

It is the argument that one of these ladies had in mind when she contemplated the using of purgatives, and yet, no doubt, holding herself to be "a very good homœopath."

It is the argument used by them to justify their intention to use alcoholic liquors upon the patients in the Hospital in direct violation of rule sixth, which says: "The use of alcoholic liquors in any form will not be allowed in the dispensary, or any department of the Hospital."

It is an error to say that physicians are restricted from using disinfectants. In these Hospitals Copperas, Platt's Chlorides, Bichloride of Mercury (or Corrosive Sublimate), Sulphate of Zinc, and roll Sulphur are used for disinfection. In the new building elaborate arrangements have been perfected for using steam-heat to disinfect clothing. The only objection made is in the case of Carbolic Acid, and that is abandoned on old-school authority, the very authority for which the malcontent doctors entertain such profound respect. The text-book used in the Hospital is *The Hand-Book for Hospitals*, published by and for the use of "The State Charities Aid Association" of New York. This work is excellent allopathic authority, and condemns Carbolic Acid in the most pointed manner. It would be well for the seceding physicians to make themselves better acquainted with the "best means known to science" for disinfection before taking such pains to champion the cause.

We deny that there is any interference with the "potencies and remedies" prescribed by the visiting physicians. The writer of this article has practiced in this same Hospital, and he was never interfered with in any manner. He did not find it necessary to use Carbolic Acid for disinfection; for in a case of uterine cancer, in which the putrescent odor was so strong as to sicken the nurses and cause one of the lady Managers to partially faint, he gave the indicated remedy, Silicea$^{2c}$, with the result of reducing the odor to an extent that surprised the Managers, several of whom were witnesses of this excellent result of applying homœopathic methods.

The sum is this: These people secure foothold in an institution that has in plain view a set of rules to which they are opposed. Notwithstanding this, they serve under these rules, and thus tacitly consent to them. They then combine to nullify them, and when remonstrated with deny the right of the authority, whose consent gained them admission, to make rules at all —a practical usurpation.

WALTER M. JAMES.

